# Isoviolanthin suppresses IL-1β-induced inflammatory and catabolic responses in chondrocytes

**DOI:** 10.3389/fphar.2026.1809512

**Published:** 2026-05-22

**Authors:** Jian-Li Yin, Min-Jun Zhao, Jia-Ying Ding, Yang-Shuo Ge, Jia-Hui Luo, Xu-Bo Wu

**Affiliations:** 1 School of Rehabilitation Science, Shanghai University of Traditional Chinese Medicine, Shanghai, China; 2 Department of Rehabilitation Medicine of Shanghai Pudong New Area People’s Hospital, Shanghai, China; 3 Department of Rehabilitation, Children’s Hospital, Zhejiang University School of Medicine, Hangzhou, Zhejiang, China

**Keywords:** extracellular matrix, inflammation, isoviolanthin, knee osteoarthritis, molecular docking, network pharmacology

## Abstract

**Background:**

Knee osteoarthritis (KOA) is characterized by the progressive degeneration of articular cartilage, necessitating novel therapeutic strategies to halt disease progression. Isoviolanthin, a natural flavonoid glycoside, has an unclear mechanism of action in KOA.

**Methods:**

We employed network pharmacology and molecular docking, supported by *in vitro* assays, to investigate the mechanisms of isoviolanthin in KOA. Predicted targets of isoviolanthin were intersected with KOA-related genes from public databases, and the resulting gene set underwent Gene Ontology and KEGG enrichment analyses, followed by molecular docking. Primary rat chondrocytes were stimulated with interleukin-1β (IL-1β) to establish an *in vitro* model of inflammation. Using a CCK-8 viability assay, we determined a working concentration of 25 μM at 24 h that did not significantly affect cell viability. The effects of isoviolanthin on inflammatory mediators, extracellular matrix metabolism, and the activation of key proteins in the PI3K-AKT and MAPK pathways were then evaluated by Western blot, RT-qPCR, and immunofluorescence.

**Results:**

Network pharmacology identified 141 overlapping targets. KEGG enrichment analysis revealed the PI3K-AKT and MAPK signaling pathways as among the most significantly enriched. Molecular docking indicated that isoviolanthin exhibits favorable binding affinities to core targets, including AKT1, MAPK1, and EGFR. In IL-1β-stimulated rat chondrocytes, 25 μM isoviolanthin significantly reduced the expression of inflammatory mediators (CXCL3, CXCL6, IL-6, iNOS, TNF-α, IL-1β, COX2) and promoted the anabolism of extracellular matrix components (collagen II, ACAN, SOX9). The treatment also suppressed matrix-degrading enzymes (MMP3, MMP9, MMP13) and attenuated the IL-1β-induced phosphorylation of PI3K-AKT and MAPK family members. Furthermore, isoviolanthin reversed the IL-1β-induced alterations in RXFP1 expression at both the mRNA and protein levels.

**Conclusion:**

These findings demonstrate that isoviolanthin exerts anti-inflammatory and ECM-protective effects in IL-1β-stimulated chondrocytes, likely by inhibiting PI3K-AKT and MAPK signaling and modulating RXFP1 expression. Further validation in human cells and *in vivo* osteoarthritis models is required to confirm target engagement and assess translational relevance.

## 1 Introduction

Knee osteoarthritis (KOA) is a prevalent chronic degenerative joint disease primarily affecting weight-bearing joints, especially the knee (Nelson, 2018). Its pathological features include progressive articular cartilage loss, subchondral bone remodeling, and synovial inflammation, collectively leading to chronic pain and functional impairment. Despite the substantial global disease burden of KOA, there are currently no treatments capable of altering the disease course. The main therapeutic approaches focus on symptomatic management, including the use of analgesics, intra-articular injections, and physical therapy, while joint replacement surgery is reserved for end-stage pathology (Zhang et al., 2016). This therapeutic gap stems from an incomplete understanding of KOA pathophysiology, highlighting the urgent need for novel targets and therapeutic agents.

The pathophysiology of KOA involves a vicious cycle of inflammation and extracellular matrix (ECM) degradation within articular cartilage. In healthy cartilage, chondrocytes maintain a matrix rich in collagen II and aggrecan proteoglycan, but in OA, these components are progressively cleaved by catabolic enzymes. Chondrocytes in osteoarthritic joints overexpress matrix metalloproteinases (MMPs) and a disintegrin and metalloproteinase with thrombospondin motifs (ADAMTS) proteases, which degrade collagen II and aggrecan (Goldring and Marcu, 2009). Concurrently, pro-inflammatory cytokines, including interleukin-1β (IL-1β) and tumor necrosis factor-α (TNF-α), are elevated in the joint microenvironment and amplify this catabolic cascade (Min et al., 2017). The chronic activation of these cytokine-mediated pathways disrupts normal repair processes, creating a feed-forward loop of ECM breakdown (Maldonado and Nam, 2013). This process ultimately leads to irreversible cartilage erosion and loss of joint function.

At the cellular level, multiple intracellular pathways transduce these inflammatory and stress signals. The phosphatidylinositol-3-kinase (PI3K)-AKT pathway, for instance, is essential for chondrocyte metabolism and survival but becomes dysregulated in OA, where it contributes to disease progression (Sun et al., 2020). The mitogen-activated protein kinase (MAPK) cascade, including its Extracellular signal-Regulated Kinases (ERK), c-Jun N-terminal kinase (JNK), and p38 branches, also serves as a key mediator of chondrocyte responses to cytokines and mechanical stress (Xu et al., 2022). Aberrant MAPK activation drives the expression of inflammatory mediators and catabolic enzymes, which directly promotes cartilage degradation (Jiang et al., 2022). Existing reviews confirm that both MAPK and PI3K-AKT signaling are critical regulators of chondrocyte catabolism, inflammation, and apoptosis in KOA (Zhang et al., 2020). Consequently, targeting these signaling nodes represents a promising strategy for correcting the inflammatory-anabolic imbalance in OA.

Given the limitations of current treatments, the potential of natural products has garnered increasing attention. In particular, plant-derived flavonoids have emerged as promising chondroprotective agents due to their anti-inflammatory and antioxidant activities (Ye and Zhou, 2023). Flavonoids such as quercetin and luteolin have been shown to alleviate IL-1β-induced inflammatory responses and ECM degradation in both cellular and animal models of osteoarthritis, primarily by inhibiting Nuclear Factor-kappa B (NF-κB), MAPK, and related signaling pathways. Thus, these compounds represent a promising source of lead molecules for the treatment of KOA (Yamaura et al., 2023; Sartinah et al., 2022).

Isoviolanthin, a flavonoid glycoside not previously investigated in KOA, is a di-C-glycoside flavone isolated from Dendrobium officinale and other medicinal plants (Xing et al., 2018; Wang et al., 2023). Unlike typical O-glycosides (e.g., isoquercitrin), its unique structure—featuring one C-linked arabinosyl moiety at C-6 and one C-linked glucosyl moiety at C-8 of the flavone core—confers greater metabolic stability, resistance to enzymatic hydrolysis, and potentially improved cellular retention and bioavailability (Xiao et al., 2016; Hostetler et al., 2017). Previous studies have demonstrated its antioxidant and cytoprotective activities, such as protecting keratinocytes from H_2_O_2_-induced damage, indicating a capacity to modulate oxidative and inflammatory stress (Wang et al., 2023). Consistent with our prior findings on the structurally related flavonoid Eupatorin (Zhao et al., 2025), we hypothesize that isoviolanthin exerts chondroprotective effects via coordinated suppression of PI3K-AKT and MAPK signaling.

To test this hypothesis, we used a network pharmacology approach integrated with molecular docking and experimental validation. Network pharmacology is a systems-level method for identifying potential molecular targets and pathways of bioactive compounds by synthesizing genomic and chemical database information (Panossian, 2025). This *in silico* analysis can reveal hub genes and enriched signaling cascades that may mediate the compound’s effects.

We performed a cross-analysis between the predicted targets of isoviolanthin and genes associated with osteoarthritis to identify key pathways for focused investigation. Given that inflammation is a critical factor in KOA, for the experimental validation, we chose to stimulate rat chondrocytes with IL-1β to establish a model for evaluating the effects of isoviolanthin. By combining these approaches, we seek to clarify the molecular pathways through which isoviolanthin exerts its cartilage-protective actions in KOA.

## 2 Materials and methods

### 2.1 Extraction and culture of primary chondrocytes

One-day-old Sprague-Dawley (SD) rats were purchased from Shanghai Sipur-Bikai Laboratory Animal Co., Ltd. (License No.: SCXK (Shanghai) 2018-0006). All animal procedures were approved by the Animal Experimental Center, Shanghai University of Traditional Chinese Medicine (approval no. PZSHUTCM2311290001) and conducted in accordance with institutional guidelines. Euthanasia was performed via an overdose of isoflurane. Subsequently, the entire body was immersed in 75% ethanol for 10 min for disinfection. Under sterile conditions in a biosafety cabinet, the tibiae and femoral condyles from both hind limbs were dissected. Articular cartilage was aseptically dissected and rinsed three times in ice-cold phosphate-buffered saline (PBS). Approximately 1 mm^3^ cartilage fragments were digested in a 0.5% collagenase solution (Sigma-Aldrich, C6885). The mixture was digested for 60 min in a 37 °C constant-temperature shaker operating at 150 rpm. After digestion, the cell suspension was centrifuged at 1,500 rpm for 5 min. The supernatant was discarded, and the pellet was resuspended in complete medium. This medium comprised Dulbecco’s Modified Eagle Medium (DMEM; Biosera, LM-D1110/500) supplemented with 10% fetal bovine serum (FBS; Biosera, FB-1058) and 1% penicillin–streptomycin (Beyotime, C0222). Finally, the cells were seeded in 10-cm culture dishes and maintained at 37 °C in a humidified incubator with 5% CO_2_. Primary chondrocytes from passages 0–2 were used for all experiments. Each independent biological replicate consisted of cells isolated from independent litters of neonatal rats (typically 4–6 pups per isolation) on different days. Cells from a single isolation were cultured and treated independently, constituting one biological replicate (n = 1).

### 2.2 Cell model construction and drug treatment

Primary articular chondrocytes were allocated to the following groups as indicated in each assay: blank control (untreated), model (IL-1β alone), and isoviolanthin intervention groups. Recombinant human IL-1β (Bio-Techne, 501-RL) was used at 20 ng/mL to induce an inflammatory phenotype. Isoviolanthin (TOPSCIENCE, T13741) was prepared as a DMSO stock solution and diluted in culture medium immediately prior to use. The final DMSO concentration in all treatments was maintained at ≤ 0.1% (v/v).

For CCK-8 assays evaluating cell viability, cells were treated with increasing concentrations of isoviolanthin (1–100 μM) without IL-1β. For other experiments involving IL-1β stimulation, cells were treated with 20 ng/mL IL-1β and the indicated concentrations of isoviolanthin. Based on preliminary viability and functional screening, 25 μM isoviolanthin (24 h co-treatment) was chosen as the working concentration for subsequent mechanistic assays. This 24 h treatment window was selected on the basis of cell viability assay data, which showed that 25 μM isoviolanthin did not induce a statistically significant reduction in chondrocyte viability at this time point (P > 0.05 vs. vehicle control; Figure 4A), reducing concern that downstream functional and mechanistic readouts were confounded by loss of cell viability. For short-term signaling assays (phospho-Western blots), cells were pretreated with 25 μM isoviolanthin for 2 h prior to stimulation with IL-1β (20 ng/mL) for 60 min, unless otherwise stated in the figure legend.

### 2.3 Cell viability assay (CCK-8)

Primary chondrocytes were seeded into 96-well plates at a density of 5 × 10^3^ cells per well in 100 μL of complete medium and incubated for 24 h to facilitate attachment. Treatments were applied as described in Section 2.2. Following 24 or 48 h of treatment, 10 μL of CCK-8 reagent (Biosharp, BS350 B) was added to each well, and the plates were incubated at 37 °C for 2 h before absorbance was measured at 450 nm using a microplate reader. Blank wells containing medium and CCK-8 without cells were included. Cell viability was calculated as

$$\text{Cell viability }\left(\%\right)=\left[\left(\text{OD}\_\text{sample}-\text{OD}\_\text{blank}\right)\;/\;\left(\text{OD}\_\text{control}-\text{OD}\_\text{blank}\right)\right]\times 100\%$$

where OD_control represents the mean optical density of vehicle control wells (cells exposed to the equivalent DMSO concentration). All experiments were conducted with three independent biological replicates, each measured in technical triplicate.

### 2.4 Measurement of cell proliferation (EdU incorporation)

Cell proliferation was assessed with the BeyoClick™ EdU assay (AF555; Beyotime, C0075). For the assay, cells were seeded in 24-well plates at a density of 1 × 10^5^ cells per well and grown to approximately 70%–80% confluence. The experimental design comprised a non-intervention group, a model group treated with 20 ng/mL IL-1β for 24 h, and five treatment groups co-exposed to 20 ng/mL IL-1β and isoviolanthin at concentrations of 1, 2.5, 10, 25, or 50 μM for 24 h. After treatment, cells were exposed to 10 μM EdU for 2 h, fixed, and permeabilized with 0.5% Triton X-100. The Click reaction was performed following the manufacturer’s instructions, and nuclei were counterstained with DAPI. Images were captured using an Olympus IX73 fluorescence microscope, and at least five random fields per well were analyzed. The proliferation index was defined as the percentage of EdU-positive nuclei relative to the total number of DAPI-stained nuclei. All experiments included three independent biological replicates.

### 2.5 Western blot

Total protein was extracted using RIPA lysis buffer (Servicebio, G2002) supplemented with protease and phosphatase inhibitors. Protein concentration was determined using a BCA assay (Thermo Fisher Scientific, 23225). Equal protein quantities (20 μg per lane) were separated by SDS-PAGE using gels prepared with the Omni-Easy™ One-Step Color PAGE Gel Kit (Epizyme, PG212) and subsequently transferred to PVDF membranes (Merck Millipore, IPVH00010) via wet transfer (100 V, 90 min) or under semi-dry conditions as indicated. Membranes were blocked for 20 min at room temperature with a rapid blocking buffer (Epizyme, PS108P), followed by overnight incubation at 4 °C with the following primary antibodies: β-actin (Beyotime, AF0003, 1:2000), Cyclooxygenase 2 (COX2, Selleck, F0327, 1:2000), IL-1β (Servicebio, GB11113, 1:2000), PI3K (Cell Signaling Technology, #4292, 1:2000), phospho-PI3K (Cell Signaling Technology, #4228, 1:2000), AKT (Cell Signaling Technology, # 4691, 1:2000), phospho-AKT (Cell Signaling Technology, #4060, 1:2000), ERK (Beyotime, AF1051, 1:2000), phospho-ERK (Beyotime, AF5902, 1:2000), JNK (Beyotime, AF1048, 1:2000), phospho-JNK (Beyotime, AF1762, 1:2000), p38 (Cell Signaling Technology, #9212, 1:2000), phospho-p38 (Cell Signaling Technology, #9215, 1:2000), RXFP1 (Affinity Biosciences, #BF8442, 1:2000) and MMP13 (Beyotime, AF7479, 1:2000). After three 5-min washes in TBST, membranes were incubated for 1 h at room temperature with HRP-conjugated secondary antibodies (anti-mouse IgG, Cell Signaling Technology, #7076, 1:7000; or anti-rabbit IgG, Cell Signaling Technology, #7074, 1:7000). Immunoreactive bands were detected by chemiluminescence using a Bio-Rad ChemiDoc system and quantified with ImageJ. For phospho-protein analysis, phosphorylated band intensities were normalized to the corresponding total protein levels (p/total), whereas target protein levels were normalized to β-actin as the loading control. All Western blot analyses were conducted with three independent biological replicates.

### 2.6 Reverse transcription–quantitative polymerase chain reaction (RT-qPCR)

Total RNA was isolated from chondrocytes using the RNAeasy™ Animal RNA Extraction Kit (Column) (Beyotime, R0024). RNA quality and concentration were determined by spectrophotometry (A260/280) and agarose gel electrophoresis. Equivalent amounts of RNA were then reverse-transcribed into cDNA using the Evo M-MLV Reverse Transcription Kit (Accurate Biology, AG11706).

Real-time qPCR was conducted using a QuantStudio 5 system (Applied Biosystems). Each 20-µL reaction mixture comprised 10 µL of SYBR Green Pro Taq HS Premix IV (Accurate Biology, Cat. AG11746), 0.5 µL each of forward and reverse primers (10 µM), 5 µL of cDNA template, and 4 µL of nuclease-free water. The thermal cycling protocol consisted of an initial denaturation at 95 °C for 30 s, followed by 40 cycles of 95 °C for 5 s and 60 °C for 30 s. Relative expression levels were determined using the 2^−ΔΔCt^ method, with β-actin serving as the normalization control. The primer sequences are listed in Table 1. Primer specificity was confirmed by melt-curve analysis, which showed a single peak for all primer pairs (Supplementary Melt Curve Plot). Although amplification efficiencies were not determined by standard curves, the primers were designed to produce short amplicons (100–200 bp), which generally allow efficient amplification under optimized conditions, supporting the use of the 2^−ΔΔCt^ method for relative quantification.

**TABLE 1:** Primer sequences used for RT-qPCR.

Gene	Primer sequences (5′-3′)
*β-Actin-F*	GCT​ACA​GCT​TCA​CCA​CCA​CA
*β-Actin-R*	GCC​ATC​TCT​TGC​TCG​AAG​TC
*IL-6-F*	CCA​CTG​CCT​TCC​CTA​CTT​CA
*IL-6-R*	TTC​TGA​CAG​TGC​ATC​ATC​GC
*CXCL3-F*	ACC​AGC​CTT​CAG​GGA​CTG​T
*CXCL3-R*	GGC​TAT​GAC​TTC​TGT​CTG​GGT
*CXCL6-F*	TTC​TGC​TGC​TGT​TCA​CAC​TG
*CXCL6-R*	TAT​CAA​CGG​AGC​TTG​TGG​GT
*ACAN-F*	GCC​TCT​CAA​GCC​CTT​GTC​TG
*ACAN-R*	GAT​CTC​ACA​CAG​GTC​CCC​TC
*ADAMTS5-F*	GAA​CAT​CGA​CCA​ACT​CTA​CTC​CG
*ADAMTS5-R*	CAA​TGC​CCA​CCG​AAC​CAT​CT
*MMP3-F*	ATG​ATG​AAC​GAT​GGA​CAG​ATG​A
*MMP3-R*	CAT​TGG​CTG​AGT​GAA​AGA​GAC​C
*MMP9-F*	GAT​CCC​CAG​AGC​GTT​ACT​CG
*MMP9-R*	GTT​GTG​GAA​ACT​CAC​ACG​CC
*TNF-α-F*	GTCGTAAACCACCAAGC
*TNF-α-R*	GACTCCAAAGTAGACCTGCCC
*RXFP1-F*	TGG​AGC​CCA​GAT​TTA​TTC​AGT​GG
*RXFP1-R*	GCC​ACA​TTT​CCA​CCA​GAA​TGA​ATG

### 2.7 Immunofluorescence analysis

Chondrocytes were seeded at a density of 4 × 10^4^ cells per well in a 24-well plate and cultured. After treatment, cells underwent three PBS washes, followed by fixation using 4% paraformaldehyde at room temperature for 10 min and subsequent permeabilization with 0.5% Triton X-100 for an equal duration. Samples were blocked for 1 h at room temperature using immunostaining blocking buffer (Beyotime, P0102), followed by overnight incubation at 4 °C with primary antibodies diluted in the same buffer. The primary antibodies employed were SRY-Box Transcription Factor 9 (SOX9, Santa Cruz, sc-20095, 1:500), Collagen II (Santa Cruz, sc-52658, 1:500), MMP13 (Santa Cruz, sc-30073, 1:500), inducible nitric oxide synthase (iNOS, Servicebio, GB11119-100, 1:500), and RXFP1 antibody (Affinity Biosciences, #BF8442, 1:500). Following three PBST washes, the samples underwent a 1-h incubation at room temperature in the dark with fluorescent secondary antibodies (goat anti-mouse Alexa Fluor 488, Invitrogen A32723, 1:1000; goat anti-rabbit Alexa Fluor 555, Invitrogen A32732, 1:1000). DAPI (Proteintech, CM07245) was applied for 10 min to visualize nuclei, with secondary antibody–only samples serving as negative controls. Images were acquired on an Olympus IX73 inverted fluorescence microscope, with identical exposure settings applied to all experimental groups. For quantification, five random fields per coverslip were imaged, and the mean fluorescence intensity was measured with ImageJ following background subtraction; the data represent the mean from three independent biological replicates.

### 2.8 Statistical analysis

Data are expressed as the mean ± standard deviation (SD). All experiments were performed with three independent biological replicates (n = 3), each derived from a separate cell isolation procedure performed on a different cohort of animals on different days. For each biological replicate, multiple wells (technical replicates) were used to obtain a within-run mean value, which was then treated as a single data point for statistical analysis. Differences among multiple groups were assessed by one-way analysis of variance (ANOVA) with Tukey’s *post hoc* test. Comparisons between two groups were performed using an unpaired two-tailed Student’s t-test. Statistical significance is indicated as follows: *P < 0.05, **P < 0.01, ***P < 0.001, ****P < 0.0001; ns denotes no significant difference (P > 0.05).

## 3 Network pharmacology analysis

### 3.1 Target screening and acquisition

#### 3.1.1 Collection of KOA disease targets

Potential disease targets associated with KOA were systematically collected by searching the following databases (all accessed on 4 August 2025) using the keyword “knee osteoarthritis” with species restricted to “*Homo sapiens*”: GeneCards (https://www.genecards.org/): Targets with a Relevance score > 1.0 were retained, to reduce the inclusion of genes with weak or uncertain disease associations, which may introduce noise. DrugBank (https://go.drugbank.com/), OMIM (https://omim.org/), and TTD (http://db.idrblab.net/ttd/): All retrieved targets were included without additional filtering, as these databases provide manually curated, high-confidence disease–gene associations. DisGeNET (https://www.disgenet.org/): For DisGeNET, the top 30 targets ranked by Gene–Disease Association score were selected to retain the most strongly supported KOA-related genes while limiting potential noise from lower-confidence associations. After integrating the search results from all databases and removing duplicates, a final set of 3,905 unique KOA-associated targets was obtained. The contribution of each database and the overlap between them are summarized in Supplementary Table S1. This comprehensive and transparent filtering strategy ensures that subsequent enrichment analyses are based on a well-defined target pool, minimizing potential bias.

#### 3.1.2 Prediction of isoviolanthin action targets

The canonical SMILES structure of the compound “Isoviolanthin” (C[C@H]1 [C@@H]([C@H]([C@H]([C@@H](O1)C2 = C(C(=C3C(=C2O)C (=O)C=C(O3)C4 = CC = C(C=C4)O)[C@H]5 [C@@H]([C@H]([C@@H]([C@H](O5)CO)O)O)O)O)O)O)O) was retrieved from the PubChem database (https://pubchem.ncbi.nlm.nih.gov, accessed on 4 August 2025) (Kim, 2016). This SMILES notation was then submitted to the PharmMapper (https://www.lilab-ecust.cn/pharmmapper/index.html, accessed on 4 August 2025) (Wang et al., 2017), Similarity ensemble approach (SEA, https://sea.bkslab.org/, accessed on 4 August 2025) (Keiser et al., 2007), and SwissTargetPrediction (http://swisstargetprediction.ch/, accessed on 4 August 2025) (Gfeller et al., 2014) online platforms to predict the potential action targets of isoviolanthin. For SwissTargetPrediction, we retained only targets with a non-zero probability score.

### 3.2 Screening of core targets and construction of protein-protein interaction (PPI) network

The collected potential target genes underwent standardized processing: using the UniProt database, gene names were uniformly converted to standard UniProt IDs. The overlap between predicted isoviolanthin targets and KOA-associated genes was determined using the VennDiagram package in R (https://www.R-project.org/, accessed on 4 August 2025), and the shared genes were designated as putative common targets. This common target set was analyzed using the STRING database (https://string-db.org, accessed on 4 August 2025), with the species parameter specified as “*Homo sapiens*” and the minimum required interaction score set to the highest confidence level (0.900); disconnected nodes were omitted from the visualization. The protein-protein interaction data were saved in TSV format.

Interaction data were imported into Cytoscape (https://www.cytoscape.org/, accessed on 4 August 2025) to visualize and construct an initial PPI network. Core modules and hub targets were then identified using the CytoNCA plugin (Tang et al., 2015) by computing node degree, betweenness, and closeness metrics. Nodes were filtered twice based on the median values of these centrality metrics, retaining only nodes whose three metrics were all above the network median, thereby forming a core target set. The CytoHubba plugin (Chin et al., 2014) was employed to identify key hub genes through the Maximal Clique Centrality algorithm, from which their densely interconnected subnetworks were subsequently isolated. For network visualization, node size was proportional to degree, while color depth indicated hub gene ranking.

### 3.3 GO functional and KEGG pathway enrichment analysis

To elucidate the biological significance of the potential core targets, this study conducted systematic functional annotation analysis on the screened intersection targets using the “ClusterProfiler” package (Yu et al., 2012) in R language. This analysis included Gene Ontology (GO) enrichment analysis and Kyoto Encyclopedia of Genes and Genomes (KEGG) pathway enrichment analysis. The GO analysis comprehensively covered three categories: Biological Process (BP), Molecular Function (MF), and Cellular Component (CC), aiming to untangle the functional characteristics of the core targets from different dimensions. The KEGG pathway analysis was employed to clarify the key signaling transduction and metabolic pathways in which these targets participate. All enrichment analyses were corrected for multiple testing using the false discovery rate (Benjamini–Hochberg) procedure, and terms/pathways with FDR-adjusted q < 0.05 were considered statistically significant (Benjamini and Hochberg, 1995; Huang et al., 2009). To focus on the most representative functions and pathways, we selected the top ten significantly enriched GO terms (ranked by q-value) and the top twenty significantly enriched KEGG pathways for in-depth interpretation. Finally, to present the analysis results intuitively and clearly, we visualized the enrichment results using an online plotting tool (http://www.bioinformatics.com.cn/, accessed on 4 August 2025).

### 3.4 Molecular docking validation

The three-dimensional structure of isoviolanthin was obtained from the PubChem database (CID: 101422758) in SDF format. The most stable conformation was generated using ChemBio3D (version 22.0.0.22) and exported as a MOL2 file. The crystal structures of the six core target proteins were downloaded from the Protein Data Bank (PDB IDs: SRC-1O42, AKT1-1H10, ESR1-1L2I, EGFR-5CNN, HSP90AA1-1YC1, MAPK1-4FV7). Using PyMOL (Version 3.1), all protein structures were prepared by removing solvent molecules and co-crystallized ligands. Receptor and ligand files were then converted to PDBQT format using AutoDockTools (Version 1.5.7).

Binding pocket selection: For each target, the docking grid was centered on the centroid of the original co-crystallized ligand after its removal, ensuring that the search space covered the experimentally validated binding site.

Grid box setup: AutoDock Vina uses a fixed grid spacing of 1.0 Å; therefore, the size_x, size_y, and size_z parameters in the configuration file directly represent the box dimensions in Angstroms. The grid center coordinates and box dimensions used for each target are provided in Supplementary Table S3.

Docking parameters: Molecular docking simulations were performed using AutoDock Vina with the following settings: exhaustiveness = 10, num_modes = 20, energy_range = 5.0 kcal/mol. The ligand was treated as fully flexible, while the receptor was kept rigid; no residue constraints or positional restraints were applied. The 20 generated binding modes were ranked by predicted binding affinity (kcal/mol), and the conformation with the lowest predicted binding energy was selected for subsequent visualization and interaction analysis.

Visualization: Binding mode visualizations were generated using PyMOL. Hydrogen bonds and key interacting residues were identified using the PyMOL distance measurement tool and are shown in Figure 3.

## 4 Results

### 4.1 Network pharmacology and molecular docking predictions

#### 4.1.1 Core targets and PPI network

**FIGURE 1:**
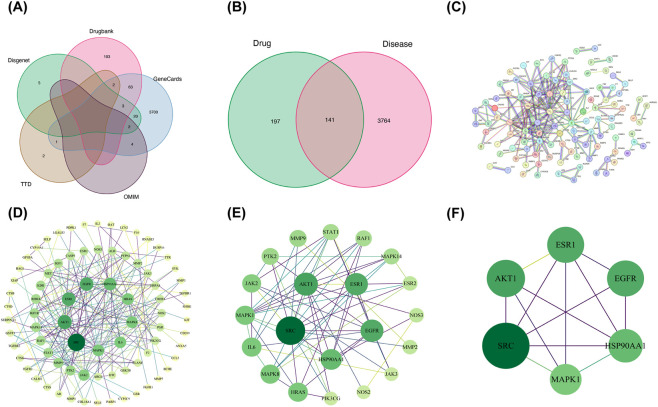
Construction of the anti-KOA target network and PPI network for isoviolanthin. **(A)** Disease targets associated with KOA were identified through integrative database mining. **(B)** A Venn diagram illustrates the overlap between targets of isoviolanthin and KOA disease targets. **(C)** A protein–protein interaction network was constructed using STRING (*Homo sapiens*, confidence score >0.9). **(D)** The workflow for iteratively screening key targets is based on network centrality metrics: Degree Centrality (DC), Closeness Centrality (CC), and Betweenness Centrality (BC). **(E)** The initial key-target set, comprising the top 21 candidates, was obtained after the first screening round. **(F)** A core target set of the top 6 candidates was derived from the second screening round, with the arrow indicating the screening workflow. DisGeNET provides curated disease–gene associations; OMIM refers to the Online Mendelian Inheritance in Man database; TTD denotes the Therapeutic Target Database; and STRING represents the Search Tool for the Retrieval of Interacting Genes/Proteins.

We identified 3,905 targets (Supplementary Data 1 KOA Disease Targets) related to KOA by integrating data from GeneCards, DrugBank, DisGeNET, TTD, and OMIM (Figure 1A). Potential targets of isoviolanthin were predicted using PharmMapper, SEA, and SwissTargetPrediction, which yielded 291, 54, and 3 candidate targets, respectively (Supplementary Data 2 Isoviolanthin Targets). Merging these results and removing duplicates produced 338 unique drug-related targets.

The intersection of these 338 drug targets with the 3,905 disease targets revealed 141 common targets, visualized in a Venn diagram (Figure 1B). These 141 intersecting targets were submitted to the STRING database (*H. sapiens*, confidence score > 0.9) to construct a PPI network of 141 nodes and 214 edges (Figure 1C); the network data were exported as a TSV file and imported into Cytoscape for further analysis. Using the CytoNCA plugin, we calculated the betweenness, closeness, and degree centrality for each node. Two rounds of iterative screening were performed using the median value of each centrality metric as a threshold: the first round identified 21 key targets (Figure 1E), and the second refined these to 6 core targets (Figure 1F) (specific thresholds are listed in Supplementary Table S2). In parallel, the CytoHubba plugin was employed to pinpoint and visualize a hub subnetwork of core genes using the maximal clique centrality algorithm.

#### 4.1.2 Enrichment analysis results

To systematically investigate the biological mechanisms of isoviolanthin in KOA, we performed GO and KEGG enrichment analyses on the 141 intersecting targets.

**FIGURE 2:**
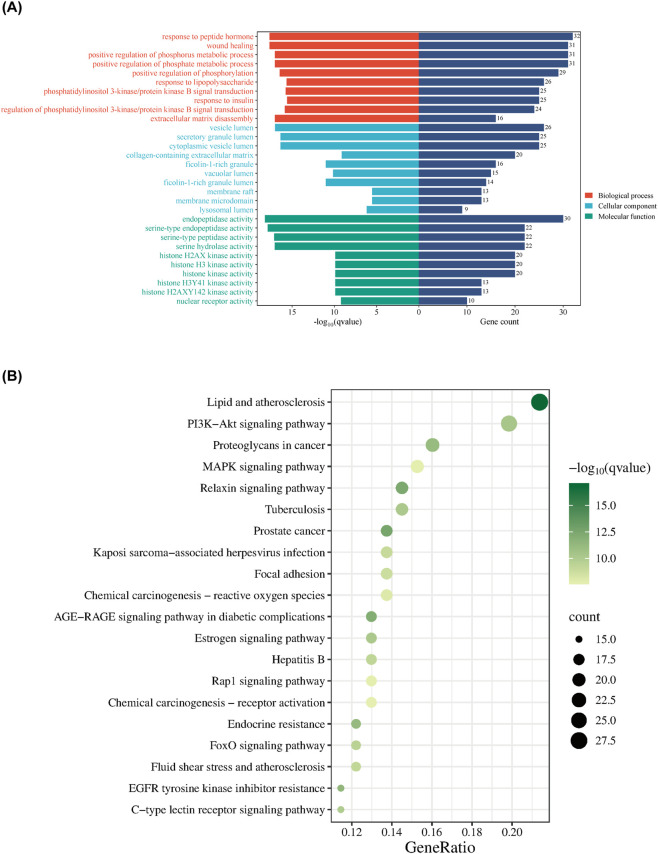
Gene Ontology (GO) and Kyoto Encyclopedia of Genes and Genomes (KEGG) enrichment analyses of the 141 intersecting targets. **(A)** Bar plot showing the top 10 significantly enriched GO terms across biological processes (BP), cellular components (CC), and molecular functions (MF). **(B)** Bubble plot illustrating the top 20 significantly enriched KEGG pathways for the same target set.

The GO analysis identified 2,265 significantly enriched terms, comprising 1,990 biological processes (BP), 47 cellular components (CC), and 228 molecular functions (MF) (Supplementary Data 3 GO). The ten most significantly enriched terms are shown in Figure 2A. For BP, the targets were primarily associated with responses to peptide hormones and lipopolysaccharides, wound healing, positive regulation of phosphorylation, phospholipid metabolic processes, insulin response, regulation of the PI3K-AKT signaling pathway, and ECM organization and degradation. Enriched CC terms mainly involved vesicle-related compartments, such as the vesicle lumen and secretory granule lumen, and the collagen-containing extracellular matrix. Key MF terms included endopeptidase activity, especially serine-type endopeptidase activity, histone kinase activity, and nuclear receptor activity.

KEGG pathway enrichment analysis revealed 164 significantly enriched pathways (q < 0.05, Supplementary Data 4 KEGG). To ensure transparency and avoid *post hoc* interpretation, pathways for experimental validation were selected based on predefined criteria: (i) statistical significance (q value), (ii) biological relevance to osteoarthritis pathology, and (iii) overlap with hub genes identified in the PPI network. Based on these criteria, the PI3K-AKT signaling pathway (q = 4.33 × 10^−11^) and MAPK signaling pathway (q = 2.04 × 10^−8^) were selected for experimental validation due to their strong statistical significance and well-established roles in chondrocyte inflammation and extracellular matrix degradation. In addition, the relaxin signaling pathway (q = 4.22 × 10^−13^), which showed the strongest statistical significance among the three pathways and contained several overlapping hub genes, prompted further exploration of its receptor RXFP1 in this study. It should be noted that pathway enrichment analysis indicates potential functional associations rather than definitive mechanistic relationships.

#### 4.1.3 Molecular docking results

**FIGURE 3:**
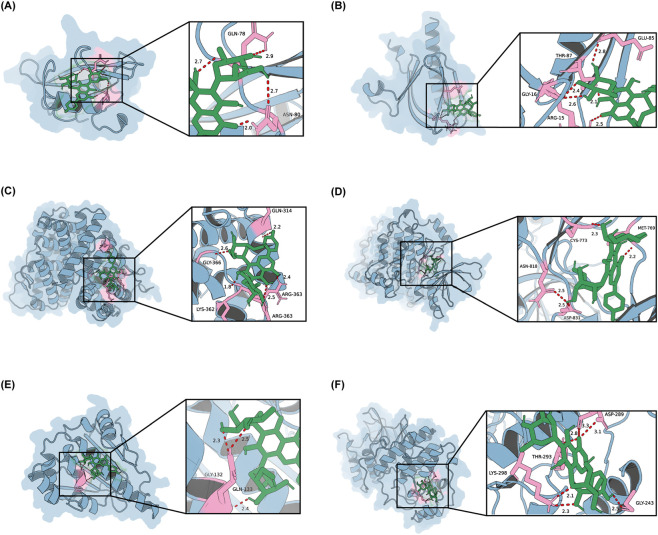
Molecular docking of isoviolanthin with six core targets. **(A)** SRC-isoviolanthin. **(B)** AKT1-isoviolanthin. **(C)** ESR1-isoviolanthin. **(D)** EGFR-isoviolanthin. **(E)** HSP90AA1-isoviolanthin. **(F)** MAPK1-isoviolanthin. For each panel, the left view illustrates the overall binding pose between isoviolanthin and the target protein, while the right view shows a magnified representation of the binding site with key interacting residues labeled. Hydrogen bonds are indicated by red dashed lines. See Methods for docking software, preparation procedure, and scoring conventions.

To further evaluate potential interactions between isoviolanthin and the six core targets identified by network analysis, we conducted molecular docking simulations. All calculated binding energies were ≤ −5.0 kcal/mol, a threshold consistent with favorable ligand–receptor binding. These values are detailed in Table 2. Figure 3 presents representative binding poses and close-up views of the key interactions, with hydrogen bonds highlighted. While these results suggest plausible direct interactions, molecular docking remains a predictive, hypothesis-generating tool rather than definitive proof of biological binding. Consequently, the docking data support the target selection but require validation through biochemical assays—such as surface plasmon resonance, isothermal titration calorimetry, or functional activity tests—to confirm affinity and functional relevance. Full methodological details, including protein and ligand preparation, scoring functions, and any refinement procedures, are provided in the Methods and Supplementary Table S3.

**TABLE 2:** Binding energies of isoviolanthin with six core protein receptors (kcal/mol).

NO.	Protein receptor	PDB id	Binding energy (kcal/mol)
1	SRC	1O42	−6.8
2	AKT1	1H10	−6.1
3	ESR1	1L2I	−7.4
4	EGFR	5CNN	−10.0
5	HSP90AA1	1YC1	−6.9
6	MAPK1	4FV7	−7.0

### 4.2 Isoviolanthin concentration screening

**FIGURE 4:**
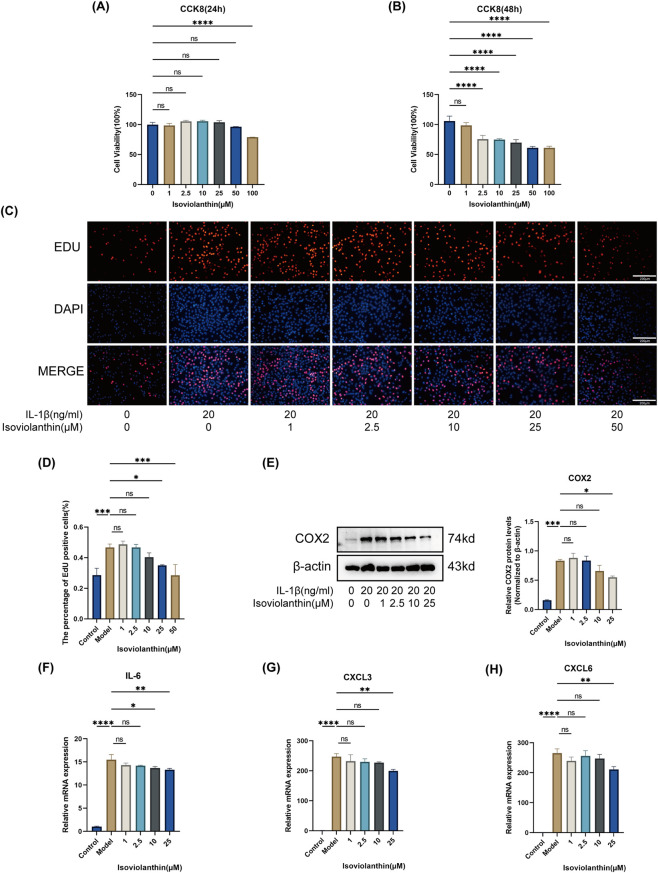
Effects of isoviolanthin on chondrocyte viability, proliferation, and inflammatory response. **(A,B)** Chondrocyte viability was assessed using the CCK-8 assay. Cells were treated with 0, 1, 2.5, 10, 25, 50, or 100 μM isoviolanthin for 24 h **(A)** or 48 h **(B)**. **(C)** Cell proliferation was evaluated by EdU staining. Chondrocytes were stimulated with 20 ng/mL IL-1β and co-treated with 1, 2.5, 10, 25, or 50 μM isoviolanthin for 24 h. Representative fluorescence images are shown; EdU-positive nuclei are red, and total nuclei (DAPI) are blue. Scale bar = 200 μm. **(D)** Quantitative analysis of the EdU-positive cell rate. **(E)** Chondrocytes were co-treated with IL-1β (20 ng/mL) and different concentrations of isoviolanthin (1, 2.5, 10, 25 μM) for 24 h. COX2 protein expression was detected by Western blot and quantified; β-actin served as the loading control. **(F–H)** mRNA expression levels of inflammatory factors were measured by RT-qPCR under the same treatment conditions in **(E)**: **(F)** IL-6, **(G)** CXCL3, **(H)** CXCL6. Data represent the mean ± SD from three independent biological replicates (n = 3), each measured in technical triplicate. Differences among multiple groups were assessed by one-way analysis of variance (ANOVA) with Tukey’s *post hoc* test. Comparisons between two groups were performed using an unpaired two-tailed Student’s t-test. Statistical significance is indicated as follows: *P < 0.05, **P < 0.01, ***P < 0.001, ****P < 0.0001; ns denotes no significant difference (P > 0.05).

CCK-8 assays revealed a time- and concentration-dependent effect of isoviolanthin on chondrocyte viability. After 24 h of treatment, only the highest tested concentration (100 μM) significantly reduced chondrocyte viability compared to the vehicle control (P < 0.0001); none of the other tested concentrations, including 1, 2.5, 10, and 25 μM, produced a statistically significant reduction in cell viability at this time point (P > 0.05 for all, Figure 4A). By 48 h, concentrations ≥2.5 μM caused a statistically significant decrease in cell viability (P < 0.0001) (Figure 4B), indicating that prolonged exposure to isoviolanthin produces time-dependent antiproliferative effects. These 48 h findings informed the decision to restrict all subsequent functional and mechanistic experiments to a 24 h treatment window, at which 25 μM isoviolanthin did not significantly affect cell viability (Figure 4A), thereby reducing concern that experimental readouts were driven by loss of cell viability. EdU incorporation assays demonstrated that IL-1β (20 ng/mL) significantly elevated the proportion of proliferating chondrocytes, while isoviolanthin co-treatment dose-dependently reduced this IL-1β-induced EdU positivity (Figures 4C,D).

Under IL-1β stimulation, we assessed the anti-inflammatory effects of isoviolanthin by measuring COX2 protein via Western blot and the mRNA levels of IL-6, C-X-C Motif Chemokine Ligand 3 (CXCL3), and C-X-C Motif Chemokine Ligand 6 (CXCL6) via RT-qPCR. IL-1β markedly upregulated COX2 protein expression and the transcription of IL-6, CXCL3, and CXCL6 (Figures 4E–H). Isoviolanthin attenuated these IL-1β-induced increases in a concentration-dependent manner, with 25 μM producing statistically significant suppression of all markers compared to IL-1β alone.

Based on the combined viability, proliferation, and inflammatory marker analyses, 25 μM was chosen as the working concentration for subsequent mechanistic experiments. This concentration did not induce a statistically significant reduction in cell viability at 24 h (Figure 4A) and effectively counteracted both IL-1β-induced inflammatory marker expression and IL-1β-driven hyperproliferation (Figures 4C–H).

### 4.3 Effects of isoviolanthin on the expression of ECM-Related proteins in IL-1β-induced chondrocytes

**FIGURE 5:**
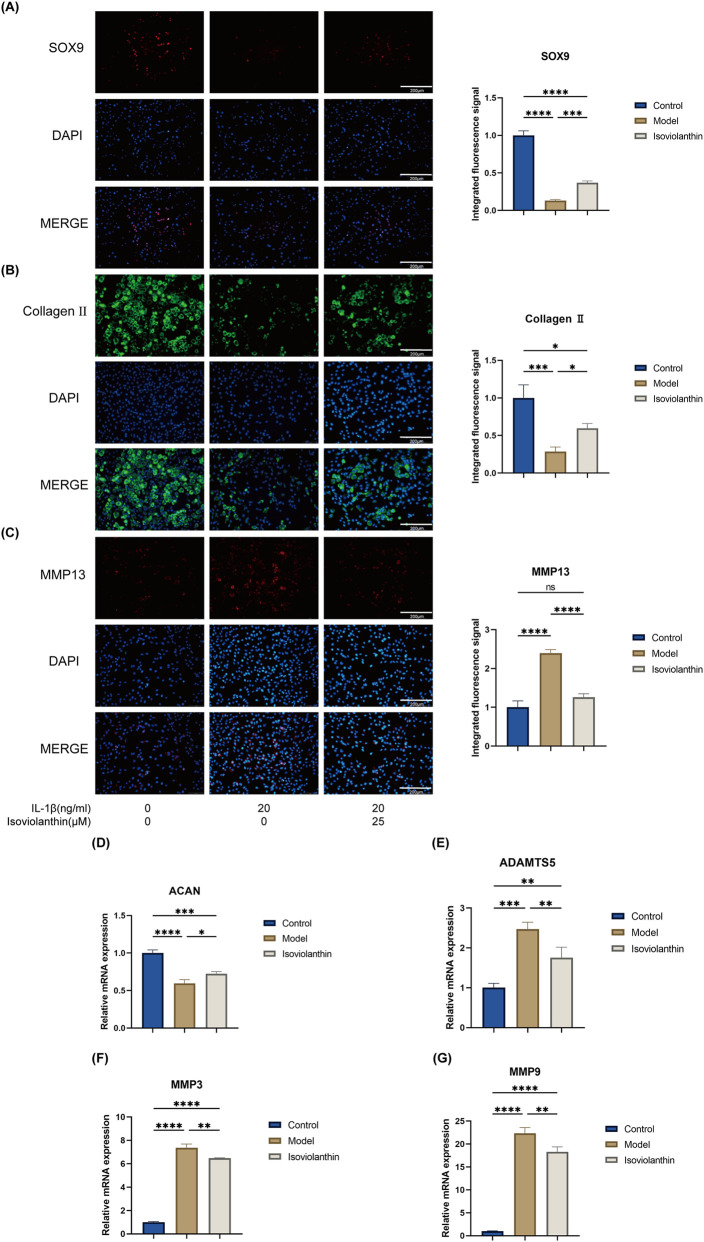
Protective effects of isoviolanthin against IL-1β-induced extracellular matrix degradation in chondrocytes. **(A–C)** Representative immunofluorescence images and corresponding quantification show SOX9 **(A)**, collagen II **(B)**, and MMP13 **(C)** expression in chondrocytes treated with IL-1β (20 ng/mL) for 24 h, either with or without 25 μM isoviolanthin. Nuclei were counterstained with DAPI. Scale bar = 200 μm. Under the same treatment regimen, RT-qPCR analysis measured mRNA expression levels of ECM-related genes **(D)** ACAN **(E)** ADAMTS5 **(F)** MMP3, and **(G)** MMP9. Data represent the mean ± SD from three independent biological replicates (n = 3), each measured in technical triplicate. Differences among multiple groups were assessed by one-way analysis of variance (ANOVA) with Tukey’s *post hoc* test. Comparisons between two groups were performed using an unpaired two-tailed Student’s t-test. Statistical significance is indicated as follows: *P < 0.05, **P < 0.01, ***P < 0.001, ****P < 0.0001; ns denotes no significant difference (P > 0.05).

Immunofluorescence analysis revealed that co-treatment with 25 μM isoviolanthin significantly increased SOX9 (P < 0.001) and collagen II (P < 0.05) protein expression, while markedly decreasing MMP13 (P < 0.0001), relative to the IL-1β model group (Figures 5A–C). These proteins were quantified from the corresponding immunofluorescence images. RT-qPCR analysis further demonstrated that isoviolanthin significantly upregulated Aggrecan (ACAN) mRNA (P < 0.05) and downregulated ADAMTS5 (P < 0.01), MMP3 (P < 0.01), and MMP9 (P < 0.01) mRNA levels compared to the IL-1β group (Figures 5D–G). Collectively, these findings indicate that isoviolanthin counteracts IL-1β-induced extracellular matrix catabolism in chondrocytes by enhancing anabolic markers (SOX9, collagen II, ACAN) and repressing catabolic enzymes (MMP13, ADAMTS5, MMP3, MMP9).

### 4.4 Isoviolanthin reduces the expression of inflammation-related proteins in IL-1β-induced chondrocytes

**FIGURE 6:**
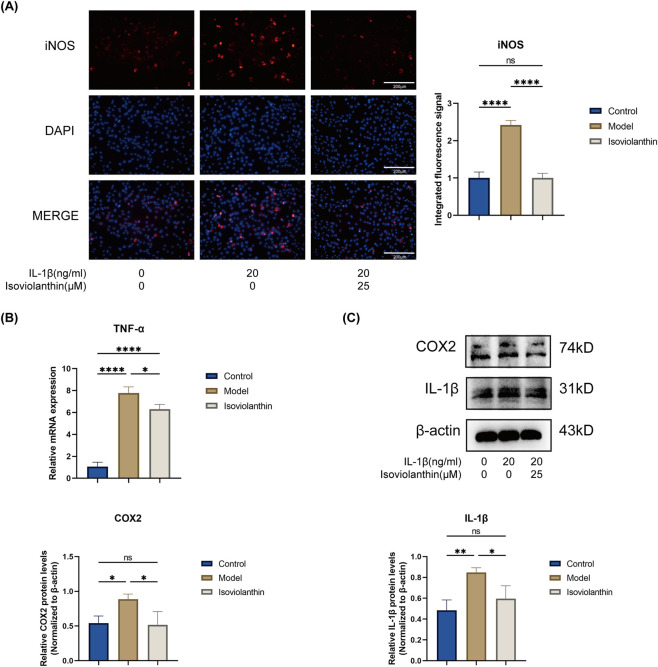
Inhibitory effects of isoviolanthin on the expression of inflammatory mediators in IL-1β-stimulated chondrocytes. **(A)** Representative immunofluorescence images and their quantification show iNOS in chondrocytes treated with or without 25 μM isoviolanthin under IL-1β (20 ng/mL) stimulation for 24 h; nuclei were stained with DAPI. Scale bar = 200 μm. **(B)** RT-qPCR measured the mRNA expression levels of TNF-α. **(C)** Western blotting was used to measure COX2 and IL-1β protein levels. Representative immunoblots and their quantitative analysis are shown, with β-actin used as a loading control. Data represent the mean ± SD from three independent biological replicates (n = 3), each measured in technical triplicate. Differences among multiple groups were assessed by one-way analysis of variance (ANOVA) with Tukey’s *post hoc* test. Comparisons between two groups were performed using an unpaired two-tailed Student’s t-test. Statistical significance is indicated as follows: *P < 0.05, **P < 0.01, ***P < 0.001, ****P < 0.0001; ns denotes no significant difference (P > 0.05).

At the established working dose of 25 μM, the impact of isoviolanthin on major inflammatory mediators was assessed in IL-1β–stimulated chondrocytes. Immunofluorescence analysis demonstrated that IL-1β (20 ng/mL) markedly increased iNOS protein expression, and this increase was significantly reduced by co-treatment with 25 μM isoviolanthin (P < 0.0001) (Figure 6A). RT-qPCR revealed that IL-1β significantly upregulated TNF-α mRNA expression, an effect attenuated by isoviolanthin (Figure 6B). Western blot analysis showed that IL-1β increased COX2 and IL-1β protein levels, while isoviolanthin co-treatment decreased these levels relative to the IL-1β group (Figure 6C). These findings together demonstrate that isoviolanthin mitigates inflammation in IL-1β–stimulated chondrocytes by lowering the expression of inflammatory markers at both the protein and mRNA levels.

### 4.5 Effects of isoviolanthin on the PI3K-AKT and MAPK signaling pathways

**FIGURE 7:**
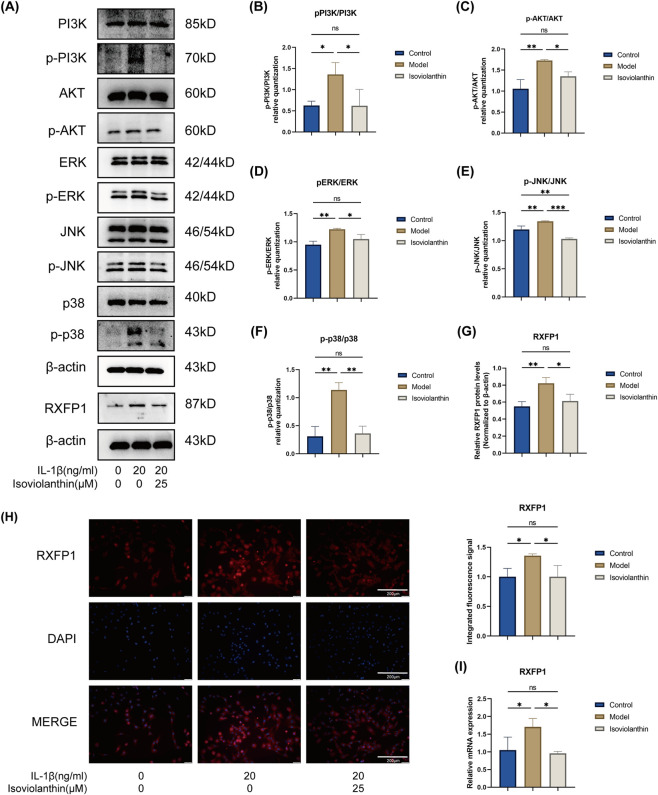
Effects of isoviolanthin on the IL-1β-induced activation of PI3K-AKT and MAPK signaling pathways and on RXFP1 expression. **(A)** Western blot analysis assessed the phosphorylation levels of key signaling proteins. Chondrocytes were pretreated with 25 μM isoviolanthin or left untreated for 2 h, then stimulated with IL-1β (20 ng/mL) for 60 min. Representative blots and their corresponding quantitative analyses are presented for p-PI3K/PI3K **(B)**, p-AKT/AKT **(C)**, p-ERK/ERK **(D)**, p-JNK/JNK **(E)**, and p-p38/p38 **(F)**. **(G)** Western blot analysis and quantification of RXFP1 protein expression in chondrocytes treated with IL-1β (20 ng/mL) for 24 h with or without 25 μM isoviolanthin. β-actin served as the loading control. **(H)** Representative immunofluorescence images and quantitative analysis of RXFP1 (green). Nuclei were stained with DAPI. Scale bar = 200 μm. **(I)** RT-qPCR analysis of RXFP1 mRNA expression under the same treatment conditions. Data represent the mean ± SD from three independent biological replicates (n = 3), each measured in technical triplicate. Differences among multiple groups were assessed by one-way analysis of variance (ANOVA) with Tukey’s *post hoc* test. Comparisons between two groups were performed using an unpaired two-tailed Student’s t-test. Statistical significance is indicated as follows: *P < 0.05, **P < 0.01, ***P < 0.001, ****P < 0.0001; ns denotes no significant difference (P > 0.05).

Guided by KEGG enrichment analysis (Figure 2B), we investigated whether isoviolanthin modulates IL-1β–induced activation of the PI3K-AKT and MAPK signaling pathways. For the detection of RXFP1 protein and mRNA expression, chondrocytes were co-treated with 25 μM isoviolanthin and IL-1β (20 ng/mL) for 24 h before harvest. For the detection of signaling pathway activation (e.g., PI3K/AKT and MAPK), chondrocytes were pretreated with 25 μM isoviolanthin for 2 h prior to stimulation with IL-1β (20 ng/mL) for 60 min. IL-1β markedly increased the phosphorylation of PI3K, AKT, ERK, JNK, and p38, as quantified by the ratios of phosphorylated to total protein (p-PI3K/PI3K, p-AKT/AKT, p-ERK/ERK, p-JNK/JNK, and p-p38/p38) (Figures 7B–F). This IL-1β-induced phosphorylation was significantly attenuated by pretreatment with 25 μM isoviolanthin (Figures 7B–F). To examine RXFP1 expression at the protein level, Western blot analysis showed that IL-1β stimulation significantly increased RXFP1 protein expression, whereas isoviolanthin co-treatment attenuated this increase (P < 0.05 vs. IL-1β) (Figure 7G). Immunofluorescence staining further confirmed these findings, showing increased RXFP1 fluorescence intensity in IL-1β-treated chondrocytes and its reduction following isoviolanthin treatment (Figure 7H). Consistent with these protein-level findings, RT-qPCR analysis showed that IL-1β significantly increased RXFP1 mRNA expression, whereas co-treatment with isoviolanthin significantly reduced this increase (P < 0.05 vs. IL-1β) (Figure 7I).

These results suggest that isoviolanthin suppresses IL-1β-induced activation of PI3K-AKT and MAPK signaling pathways and modulates RXFP1 expression at both the mRNA and protein levels, which may contribute to its chondroprotective effects.

**FIGURE 8:**
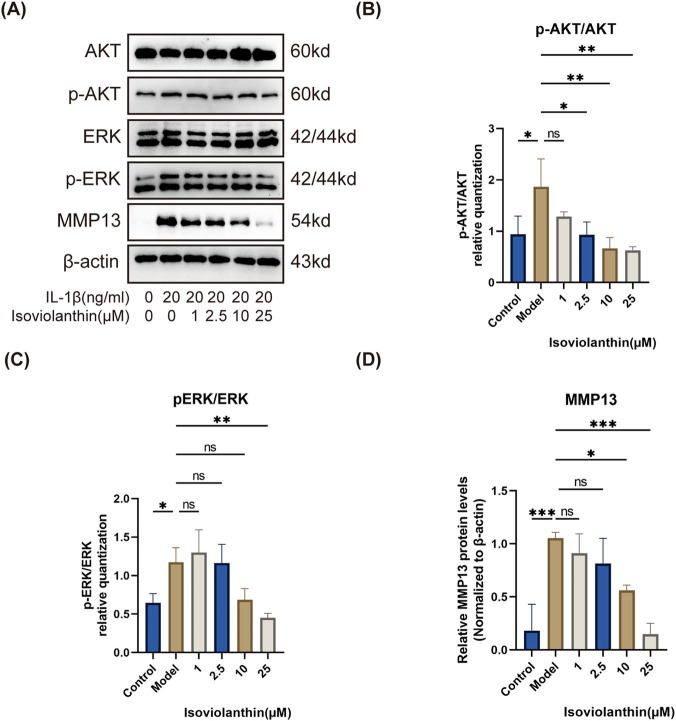
Concentration-dependent effects of isoviolanthin on p-AKT/AKT, p-ERK/ERK, and MMP13 protein expression in IL-1β-stimulated chondrocytes. **(A)** Representative Western blot images showing protein bands for p-AKT, total AKT, p-ERK, total ERK, MMP13, and β-actin across treatment groups (Control, IL-1β, IL-1β + 1 μM, IL-1β + 2.5 µM, IL-1β + 10 μM, IL-1β + 25 µM isoviolanthin). Quantitative analyses of **(B)** p-AKT/AKT **(C)** p-ERK/ERK, and **(D)** MMP13/β-actin ratios. For panels B and C, chondrocytes were pretreated with isoviolanthin at the indicated concentrations for 2 h prior to stimulation with IL-1β (20 ng/mL) for 60 min. For panel D, chondrocytes were co-treated with IL-1β (20 ng/mL) and isoviolanthin at the indicated concentrations for 24 h β-actin served as the loading control for MMP13 quantification; phosphorylated protein levels were normalized to the corresponding total protein levels. Data represent the mean ± SD from three independent biological replicates (n = 3), each measured in technical triplicate. Differences among multiple groups were assessed by one-way analysis of variance (ANOVA) with Tukey’s *post hoc* test. Comparisons between two groups were performed using an unpaired two-tailed Student’s t-test. Statistical significance is indicated as follows: *P < 0.05, **P < 0.01, ***P < 0.001; ns denotes no significant difference (P > 0.05).

To further evaluate whether the effects of isoviolanthin on PI3K-AKT/MAPK signaling and downstream catabolic markers are concentration-dependent, Western blot analyses of p-AKT/AKT, p-ERK/ERK, and MMP13 were performed across 1–25 µM isoviolanthin (Figure 8). For signaling proteins (p-AKT, p-ERK), chondrocytes were pretreated with isoviolanthin for 2 h prior to IL-1β stimulation (20 ng/mL, 60 min). For MMP13, chondrocytes were co-treated with IL-1β and isoviolanthin for 24 h. p-AKT/AKT showed concentration-dependent suppression with significant inhibition at 2.5 µM (P < 0.05), 10 µM (P < 0.01), and 25 µM (P < 0.01) relative to the IL-1β group. p-ERK/ERK showed no significant change at lower concentrations, reaching significant suppression at 25 µM (P < 0.01); overall, a decreasing trend was observed at higher concentrations relative to the IL-1β group. MMP13 protein expression was reduced in a concentration-dependent manner, with significant effects at 10 µM (P < 0.05) and 25 µM (P < 0.001), linking the dose-dependent signaling changes to a functionally relevant downstream catabolic endpoint.

## 5 Discussion

In this study, we combined network pharmacology, molecular docking and *in vitro* experiments to investigate the mechanisms by which the flavonoid glycoside isoviolanthin modulates key processes relevant to KOA. Using an IL-1β–stimulated primary rat chondrocyte model, we found that co-treatment with 25 μM isoviolanthin attenuated the IL-1β–driven inflammatory response (reduced CXCL3, CXCL6, TNF-α, IL-1β, IL-6 and COX2), restored markers of ECM anabolism (increased SOX9, collagen II and ACAN) and suppressed catabolic enzymes (decreased MMP3, MMP9 and MMP13). Mechanistically, isoviolanthin inhibited IL-1β-induced phosphorylation of PI3K, AKT and MAPK family members (ERK, JNK, p38), and reversed IL-1β–induced upregulation of RXFP1 expression at both the mRNA and protein levels. The *in vitro* experimental data align with the network pharmacology and molecular docking analyses, reinforcing the concept that isoviolanthin exerts its chondroprotective effects through a multi-target and multi-pathway mechanism.

The present findings align with several aspects of established flavonoid pharmacology. Notably, our previous study (Zhao et al., 2025) demonstrated that the flavonoid Eupatorin exerts chondroprotective effects in osteoarthritis via modulation of the PI3K-AKT and estrogen signaling pathways. Plant flavonoids, such as quercetin (Yamaura et al., 2023) and luteolin (Sartinah et al., 2022), are widely reported to suppress NF-κB, PI3K-AKT, and MAPK signaling, thereby exerting anti-inflammatory and matrix-protective effects in both cellular and animal models of OA. Our data extend this class effect to isoviolanthin, demonstrating that its inhibition of PI3K-AKT and MAPK pathways correlates with reduced transcription and protein expression of inflammatory mediators and matrix-degrading enzymes in chondrocytes. Furthermore, Xing et al. (Xing et al., 2018) previously reported that isoviolanthin modulates MMP expression and PI3K-AKT-related signaling in other cell types, providing indirect yet supportive precedent for the mechanisms we observed.

To clarify its mechanism of action, this research prioritized the validation of the PI3K-AKT and MAPK signaling cascades, as these pathways were prominently identified through network pharmacology-based enrichment analysis. In classical OA pathological models, IL-1β promotes the expression of inflammatory mediators and MMPs by activating pathways such as NF-κB and MAPK (Liacini et al., 2002; Choi et al., 2013). We found that isoviolanthin significantly inhibited IL-1β-induced phosphorylation of PI3K, AKT, JNK, ERK1/2, and p38 in chondrocytes. Extensive crosstalk exists between the PI3K-AKT and MAPK cascades, with documented bidirectional regulatory interactions in cellular stress and inflammatory responses (Zhou et al., 2015; Cao et al., 2019). The PI3K-AKT signaling pathway is crucial for regulating chondrocyte metabolism and can influence inflammatory processes, connecting it to transcription dependent on NF-κB (Sun et al., 2020). Thus, the simultaneous suppression of both PI3K-AKT and MAPK pathways by isoviolanthin is expected to diminish the function of key transcription factors, including AP-1 and NF-κB. This leads to a broad transcriptional suppression of inflammatory cytokines and MMPs, which explains its multi-faceted chondroprotective effects (Liacini et al., 2002).

It should be noted that the core targets predicted by network pharmacology in this study—including AKT1 and MAPK1—have not been directly validated through biochemical binding assays such as surface plasmon resonance or isothermal titration calorimetry. However, functional cell-based evidence supporting the involvement of these targets is provided by the concentration-dependent suppression of p-AKT/AKT and p-ERK/ERK ratios across 1–25 µM isoviolanthin (Figure 8; see Discussion paragraph on working concentration below for quantitative details). The concentration-dependent nature of this phosphorylation inhibition provides pathway-level functional evidence consistent with AKT1 and MAPK1 involvement in the chondroprotective effects of isoviolanthin, and is more consistent with pathway-level pharmacological modulation than with purely non-specific cellular interference. Direct confirmation of molecular binding between isoviolanthin and its predicted targets remains a critical priority for future studies, as outlined in the follow-up research plan below.

The working concentration of 25 μM warrants further discussion in light of concerns regarding pleiotropic effects. To directly address whether the observed multi-pathway modulation reflects specific pharmacological activity or non-specific cellular interference, we examined the concentration-dependence of PI3K-AKT and MAPK pathway suppression, and the downstream catabolic marker MMP13, across 1, 2.5, 10, and 25 µM isoviolanthin in IL-1β-stimulated chondrocytes (Figure 8). p-AKT/AKT showed statistically significant concentration-dependent suppression from 2.5 µM onward (P < 0.05 at 2.5 µM; P < 0.01 at 10 and 25 µM), while p-ERK/ERK reached significant suppression at 25 µM (P < 0.01), indicating that ERK inhibition requires a higher isoviolanthin concentration than AKT inhibition. This differential sensitivity is consistent with the distinct upstream regulatory mechanisms of PI3K-AKT and MAPK cascades, and is more consistent with pathway-level pharmacological modulation than with non-specific cellular interference as the primary explanation. Furthermore, MMP13 protein expression was reduced in a concentration-dependent manner, with significant effects observed at 10 µM (P < 0.05) and 25 µM (P < 0.001), linking the dose-dependent signaling changes to a functionally relevant catabolic endpoint. This graded pharmacological response across multiple independent readouts argues against a purely threshold or non-specific mechanism, and supports the interpretation that 25 µM represents the upper end of the tested active range under the experimental conditions of this study. This concentration is also consistent with those employed for structurally related flavonoid C-glycosides, including vitexin and orientin, in chondrocyte inflammation models (10–50 µM) (Xie et al., 2018; Chen et al., 2025), and aligns with our prior study on eupatorin in the same experimental system (Zhao et al., 2025).

An unexpected finding of this study was the increase in EdU-positive chondrocytes following 24 h IL-1β stimulation (Figures 4C,D). While IL-1β is typically associated with catabolic and pro-apoptotic effects in OA, acute exposure to IL-1β has been shown to induce transient chondrocyte proliferation *in vitro*, possibly as an early stress response (Simsa-Maziel and Monsonego-Ornan, 2012; Iwamoto et al., 1989). This initial hyperproliferation phase may precede the shift toward catabolism and apoptosis under chronic inflammatory conditions. The reversal of this effect by isoviolanthin further supports its modulatory role in chondrocyte homeostasis.

A notable finding was the regulation of RXFP1. Network enrichment analysis linked the relaxin signaling pathway to predicted isoviolanthin targets. Experimentally, we observed that IL-1β significantly increased RXFP1 expression at both the mRNA and protein levels, whereas isoviolanthin treatment partially reversed this upregulation. Western blot and immunofluorescence analyses further confirmed these changes at the protein level, supporting the reliability of the transcriptional findings. RXFP1, the canonical receptor for relaxin peptides, has been reported to regulate extracellular matrix remodeling and MMP expression through downstream effectors including PI3K-AKT and MAPK signaling pathways (Ahmad et al., 2012). However, the functional role of relaxin signaling in cartilage and connective tissues appears to be context-dependent. In certain models, RXFP1 activation promotes matrix degradation by inducing MMP expression, whereas in other settings relaxin signaling exerts anti-fibrotic or anti-inflammatory effects. These divergent observations likely reflect differences in ligand isoforms, receptor expression levels, cell types, and microenvironmental conditions (Fan et al., 2025; Sarwar et al., 2017; Wieczfinska and Pawliczak, 2022). Therefore, the IL-1β-induced upregulation of RXFP1 observed in this study may represent an inflammation-associated response that predisposes chondrocytes to enhanced catabolic signaling. The ability of isoviolanthin to suppress RXFP1 expression suggests that modulation of relaxin signaling could contribute to its protective effects. Nevertheless, the present data remain correlative, and additional studies involving genetic or pharmacological manipulation of RXFP1 will be necessary to determine whether this receptor directly mediates the downstream PI3K-AKT/MAPK signaling changes induced by isoviolanthin.

This study also offers a broader view of the molecular regulatory network in KOA. Beyond the validated pathways, enrichment analysis indicates that isoviolanthin may influence additional signaling cascades, including FoxO, VEGF, and IL-17 (Supplementary Data 4 KEGG). FoxO transcription factors, especially FoxO3a, are crucial for chondrocyte redox homeostasis and autophagy, with established roles in both the pathogenesis and protection of osteoarthritic cartilage (Akasaki et al., 2014; Zhao et al., 2022; Wu et al., 2025). For instance, inhibiting PI3K-AKT signaling can alleviate AKT-mediated suppression of FoxO activity, thereby promoting FoxO-driven antioxidant responses and autophagy that protect chondrocytes from stress and potentially slow matrix degradation (Huang et al., 2026; Xue et al., 2017). MAPK signaling occupies an upstream position capable of cross-regulating cytokine and angiogenic pathways: IL-17 signaling activates MAPK effectors (p38, JNK, ERK) to induce inflammatory mediators, and this IL-17–MAPK crosstalk contributes to cartilage catabolism in OA (Suyama et al., 2022; Xiao et al., 2023). VEGF signaling is similarly connected to MAPK activation in cartilage and synovium, promoting angiogenesis and pathological remodeling associated with disease progression (Jiang et al., 2016; Qian et al., 2021). Consequently, by targeting a pivotal regulatory node like PI3K-AKT/MAPK, isoviolanthin may exert extensive downstream network effects—relieving the suppression of protective transcriptional programs (FoxO/autophagy) and modulating inflammatory-angiogenic axes (IL-17, VEGF)—thereby comprehensively influencing chondrocyte inflammation, metabolism, stress responses, and matrix homeostasis (Figure 2; Supplementary Data 4 KEGG) (Batarfi et al., 2025).

This work has several important limitations that should be considered when interpreting the findings. First, regarding the working concentration of isoviolanthin: the 25 μM dose used in our mechanistic assays, while consistent with concentrations widely employed for structurally related flavonoid C-glycosides (e.g., vitexin, orientin) in *in vitro* chondrocyte inflammation models, is relatively high. The newly added concentration-gradient data (Figure 8) show that significant AKT inhibition is detectable from 2.5 µM and significant MMP13 suppression from 10 μM, suggesting that 25 µM represents the upper end of the tested active range under the experimental conditions of this study rather than an arbitrary supra-pharmacological dose. Nevertheless, further studies using lower concentration ranges are warranted to define the minimum effective concentration across all endpoints and to confirm that the observed pathway modulation is retained at doses with a wider therapeutic window.

Second, regarding dose-response characterization: dose-response data are now available for cell viability, proliferation, key inflammatory markers (COX2, IL-6, CXCL3, CXCL6), core signaling pathway markers (p-AKT/AKT and p-ERK/ERK), and the downstream catabolic marker MMP13, across concentrations spanning 1–25 μM, together providing stronger evidence of concentration-dependent activity across key functional and mechanistic endpoints (Figures 4, 8). However, ECM anabolic markers (SOX9, collagen II, ACAN), additional inflammatory mediators (iNOS, TNF-α, IL-1β protein), and RXFP1 expression remain characterized at the single working concentration of 25 µM. Comprehensive dose-response characterization of these remaining endpoints, together with time-course studies, is listed as a priority for future work.

Third, regarding cytotoxicity and long-term antiproliferative activity: the mechanistic experiments in this study were conducted under treatment conditions at which 25 µM isoviolanthin did not significantly affect cell viability. Specifically, ECM markers, inflammatory mediators, and RXFP1 expression were assessed under 24 h co-treatment conditions, at which 25 μM isoviolanthin did not induce a statistically significant reduction in chondrocyte viability (P > 0.05 vs. vehicle control, Figure 4A), thereby reducing concern that cell death confounded these experimental readouts. The PI3K-AKT/MAPK signaling assays employed a substantially shorter treatment window (2 h pretreatment +60 min stimulation), further reducing concern about cytotoxicity-related interference. This 24 h window was selected specifically to avoid the time-dependent antiproliferative effects observed at 48 h. However, statistically significant reductions in cell viability were observed at concentrations ≥2.5 μM following 48 h of treatment (P < 0.0001, Figure 4B), indicating that prolonged exposure to isoviolanthin produces time-dependent antiproliferative effects. We acknowledge that the long-term cytotoxic or antiproliferative potential of isoviolanthin cannot be fully excluded from the present data, and that this has implications for translational development. Future studies should (1): characterize the mechanism underlying reduced viability at 48 h through dedicated cell cycle, apoptosis, and necrosis assays, given that CCK-8 measures mitochondrial metabolic activity rather than cell number directly (2); conduct comprehensive dose–response and time-course experiments to precisely define the therapeutic window; and (3) perform *in vivo* pharmacokinetic and toxicological studies to evaluate whether pharmacologically active concentrations can be achieved safely in target joint tissues without systemic or local toxicity.

Fourth, a separate vehicle control group was not included in the experimental design. Although the final DMSO concentration in all treatments was maintained at ≤ 0.1%, which is generally considered to have minimal biological effects in chondrocytes, future experiments should include a dedicated vehicle control (0.1% DMSO) to further exclude potential solvent-related effects.

Fifth, the experiments were conducted using neonatal rat chondrocytes in an *in vitro* IL-1β-stimulated chondrocyte inflammation model. While this model reproduces key inflammatory and catabolic features of osteoarthritis, it cannot fully recapitulate the complex cellular interactions, biomechanical loading, and tissue-level crosstalk present in the intact joint. Moreover, neonatal rat chondrocytes may differ phenotypically from adult human OA chondrocytes, limiting direct extrapolation to the clinical context. Therefore, the current findings should be considered as *in vitro* proof-of-concept and require further validation in human chondrocytes, cartilage explants, and established animal models of OA (e.g., surgical DMM or chemical MIA models) to evaluate therapeutic efficacy and translational relevance.

Sixth, while concentration-dependent suppression of p-AKT/AKT and p-ERK/ERK provides functional cell-based evidence consistent with involvement of the core predicted targets AKT1 and MAPK1 (Figure 8), direct biochemical binding validation has not been performed. Techniques such as surface plasmon resonance or isothermal titration calorimetry will be necessary to confirm direct molecular interaction between isoviolanthin and its predicted targets, particularly AKT1 and MAPK1, as well as other predicted targets including EGFR; kinase activity assays will additionally be useful to assess target-related functional inhibition.

Seventh, mechanistic validation in this study focused primarily on PI3K-AKT and MAPK signaling, whereas other enriched pathways identified by network pharmacology, including NF-κB, FoxO, and IL-17 signaling, remain to be experimentally investigated.

Eighth, although RXFP1 expression was regulated by isoviolanthin at both the mRNA and protein levels, the functional significance of this change and whether RXFP1 directly mediates the chondroprotective effects of isoviolanthin require further investigation through genetic perturbation (e.g., siRNA/shRNA knockdown) or pharmacological modulation approaches.

To address these limitations and strengthen the mechanistic and translational evidence, we propose the following prioritized follow-up studies: (1) Biochemical target validation: confirm direct binding between isoviolanthin and high-priority predicted targets (e.g., AKT1, MAPK1, EGFR) using surface plasmon resonance, isothermal titration calorimetry, or enzyme-activity assays to obtain affinity and kinetic parameters; (2) RXFP1 functional interrogation: perform genetic perturbation (siRNA/shRNA or CRISPR/Cas9) and/or pharmacological modulation of RXFP1 and evaluate effects on downstream PI3K-AKT/MAPK activation and MMP expression to test causality; (3) Pathway rescue strategies: using pathway-specific activators (e.g., IGF-1 for PI3K-AKT) or constitutively active AKT/MEK constructs to determine whether reactivation of these pathways can reverse the protective effects of isoviolanthin, thereby clarifying whether inhibition of PI3K-AKT/MAPK signaling is required for its chondroprotective actions; (4) Expanded pharmacology: conduct dose–response and time-course studies to define potency, efficacy, and treatment windows, and perform preliminary ADME/Tox characterization; (5) *In vivo* efficacy and safety: evaluate therapeutic potential in established OA models (e.g., surgical DMM or MIA models), including histopathology, pain/functional readouts, pharmacokinetics, and toxicology and (6) Human tissue validation: confirm key molecular and phenotypic effects in human cartilage explants or primary human chondrocytes to assess translational potential.

Implementing these studies—beginning with biochemical binding assays and RXFP1 functional tests, followed by pathway rescue experiments, expanded pharmacology, and *in vivo* validation—will clarify target engagement, define the mechanism of action, and generate the preclinical evidence required to further evaluate isoviolanthin as a potential therapeutic agent for KOA.

## 6 Conclusion

In conclusion, our integrated approach demonstrates that isoviolanthin mitigates IL-1β-induced inflammatory and catabolic responses in chondrocytes. These findings provide a mechanistic rationale for further exploring isoviolanthin as a potential therapeutic agent for KOA. However, as discussed above, validation in human chondrocytes and *in vivo* osteoarthritis models will be necessary before conclusions regarding disease-modifying potential can be drawn, and additional biochemical, genetic, and pharmacological studies will be required to confirm target engagement, therapeutic efficacy, and safety.

## Data Availability

All relevant data presented in the study are included in the article/Supplementary Material, further inquiries can be directed to the corresponding author.
