# Supplementary material for: Isoviolanthin suppresses IL-1β-induced inflammatory and catabolic responses in chondrocytes
Source: Front Pharmacol. 2026 May 22;17:1809512. doi: 10.3389/fphar.2026.1809512 (PMC13237640; doi:10.3389/fphar.2026.1809512)
Supplement: Supplementary file 1 [file Supplementaryfile1.docx]

Supplementary Material

**Supplementary Table 1** **Composition of KOA-associated targets from multiple databases**

| **Database** | **Retrieved Targets**  **(before deduplication)** | **Unique Contribution**  **(after deduplication)** | **Notes** |
| --- | --- | --- | --- |
| **GeneCards** | **4293** | **3795** | **Relevance score > 1.0** |
| **DrugBank** | **171** | **171** | **All included** |
| **OMIM** | **6** | **6** | **All included** |
| **TTD** | **5** | **5** | **All included** |
| **DisGeNET** | **30** | **30** | **Top 30 by association score** |
| **Total (unique)** | - | **3905** | **After removing inter-database duplicates** |

**Supplementary Table 2 Centrality metrics and screening thresholds for PPI network nodes**

| **NO.** | **BC** | **CC** | **DC** | **Eigenvector** | **LAC** | **Network** |
| --- | --- | --- | --- | --- | --- | --- |
| **1** | **17.34206349** | **0.3** | **3** | **0.035877127** | **1** | **2** |
| **2** | **308.5870962** | **0.376811594** | **9** | **0.179722533** | **3** | **4.314285714** |

NOTE: The screening criteria were the median.

**Supplementary Table 3 The Binding Energy of the Compound and Hub Targets (kcal/mol)**

| **NO.** | **Protein Receptors** | **PDB ID** | **Binding Energy (kcal/mol)** | **Grid Box Center** | | | **Size** | | | **grid spacing (Å)** | **Binding Pocket Source** |
| --- | --- | --- | --- | --- | --- | --- | --- | --- | --- | --- | --- |
|  |  |  |  | **X** | **Y** | **Z** | **X** | **Y** | **Z** |  |  |
| 1 | SRC | 1O42 | -6.8 | 10.562 | 20.172 | 21.652 | 82 | 82 | 126 | 1.0 | Co-crystallized ligand |
| 2 | AKT1 | 1H10 | -6.1 | 23.515 | 14.628 | 8.936 | 100 | 90 | 118 | 1.0 | Co-crystallized ligand |
| 3 | ESR1 | 1L2I | -7.4 | 9.653 | 0.423 | 7.711 | 126 | 126 | 124 | 1.0 | Co-crystallized ligand |
| 4 | EGFR | 5CNN | -10.0 | 33.818 | 10.403 | -19.174 | 106 | 126 | 112 | 1.0 | Co-crystallized ligand |
| 5 | HSP90AA1 | 1YC1 | -6.9 | 0.919 | 32.151 | -0.994 | 126 | 126 | 126 | 1.0 | Co-crystallized ligand |
| 6 | MAPK1 | 4FV7 | -7.0 | 3.868 | -1.639 | 11.763 | 126 | 126 | 126 | 1.0 | Co-crystallized ligand |

**Note**: Grid coordinates are in Å units relative to the PDB structure coordinate system. All docking runs used AutoDock Vina with exhaustiveness = 10.
